# Case Report: Severe Peripartum Cardiac Disease in Myotonic Dystrophy Type 1

**DOI:** 10.3389/fcvm.2022.899606

**Published:** 2022-06-03

**Authors:** Georgia Besant, Pierre R. Bourque, Ian C. Smith, Sharon Chih, Mariana M. Lamacie, Ari Breiner, Jocelyn Zwicker, Hanns Lochmüller, Jodi Warman-Chardon

**Affiliations:** ^1^Faculty of Medicine, University of Ottawa, Ottawa, ON, Canada; ^2^Department of Medicine, The Ottawa Hospital, University of Ottawa, Ottawa, ON, Canada; ^3^Ottawa Hospital Research Institute, Ottawa, ON, Canada; ^4^University of Ottawa Heart Institute, Ottawa, ON, Canada; ^5^Children’s Hospital of Eastern Ontario Research Institute, Ottawa, ON, Canada

**Keywords:** pregnancy, cardiomyopathy, spontaneous coronary artery dissection, neuromuscular disease, cardiovascular

## Abstract

**Background:**

Myotonic dystrophy type 1 (DM1) is a hereditary muscular dystrophy affecting ∼2.1–14.3/100,000 adults. Cardiac manifestations of DM1 include conduction disorders and rarely cardiomyopathies. DM1 increases the risk of obstetric complications, however, little is known about the relationship between pregnancy and cardiomyopathy in DM1 due to disease rarity.

**Case:**

A 23-year-old with DM1 developed cardiomyopathy during pregnancy. Despite initial medical stabilization, she subsequently developed multiple spontaneous coronary artery dissections postpartum, worsening cardiomyopathy and multiorgan failure. She died 5 months postpartum.

**Conclusion:**

Though cardiomyopathy and arterial dissection are both known complications of pregnancy, this case suggests individuals with myotonic dystrophy type 1 may be at heightened risk for cardiac disease during the peripartum period. Physicians caring for women with suspected or proven DM1 should offer counseling and be alerted to the risk of cardiac complications with pregnancy and in the peripartum period. Pregnant and peripartum women with DM1 are likely to benefit from more frequent assessments of cardiac function including echocardiograms and early institution of heart failure management protocols when symptoms of cardiomyopathy present.

## Introduction

Myotonic dystrophy type 1 (DM1) is one of the most common inherited muscular dystrophies in adults, with a worldwide prevalence of 2.1–14.3/100,000 (1). It is an autosomal dominant disorder, caused by the expansion of a trinucleotide (CTG) repeat sequence in the 3′ untranslated region of the myotonic dystrophy protein kinase gene (*DMPK*), located on chromosome 19q13.32 (2, 3). DM1 can present at any age and the clinical phenotype ranges from asymptomatic to severe congenital disease. Classic DM1 presents with facial and distal muscle weakness, myotonia, and cataracts in adults. DM1 patients may also have serious systemic manifestations, including central nervous system involvement, cardiac arrhythmias, and gastrointestinal disorders (4). Currently, there are no approved genetic therapies for DM1.

Cardiac involvement in DM1 is known to increase the risk of sudden cardiac death (5). Cardiac abnormalities in DM1 include conduction defects (atrioventricular block, bundle branch blocks, and intraventricular block), arrhythmias (supraventricular or ventricular tachyarrhythmias) and less commonly, cardiomyopathy and valvular disease (5–7). The most prevalent cardiac defects are conduction abnormalities, which occur in approximately 65% of patients (4). Cardiac muscle myotonia or fibrosis may contribute to left ventricular diastolic dysfunction (8). The prevalence of left ventricular systolic dysfunction in DM1 patients ranges between 7.2 and 18.9% (5, 9, 10), however most DM1 patients do not exhibit symptoms of heart failure. Both left ventricular systolic dysfunction and heart failure are significantly associated with all-cause death and cardiac death (9).

Women with DM1 are at risk of complications during pregnancy, including increased need for caesarian section (31–36.7%), pre-term labor (30–35.0%), polyhydramnios (10–25%), miscarriage (12–15.3%), urinary tract infection (9.4%), pre-eclampsia (9–9.5%), placenta previa (4–10.8%), and ectopic pregnancy (3.5–4%) (11–13). The frequency of perinatal mortality ranges from 10 to 23%, compared to 0.5–1% in the general population (11). Select symptoms of DM1, including myotonia, mobility limitations, fatigue and pain may progress during pregnancy and in some cases may not return to baseline until 6 months after pregnancy (13). In addition, many women are unaware that they are affected with DM1 before they become pregnant and may be diagnosed after their affected child displays DM1 symptoms (11).

One previous case report described a patient with DM1 who developed cardiomyopathy and died of a cardiac arrest 8 weeks postpartum (14). The second comparable observation we report here, with the additional novel finding of multiple spontaneous coronary arterial dissections, increases the likelihood of a true etiological link between DM1 and cardiac complications of pregnancy.

## Case

**FIGURE 1:**
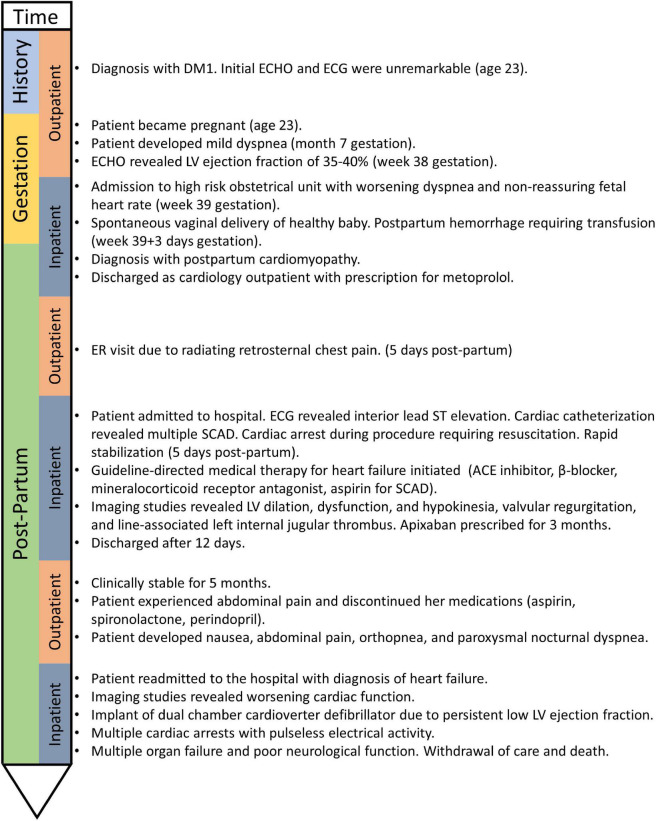
Case timeline.

A 23-year-old woman was referred to the Neuromuscular Clinic at The Ottawa Hospital for assessment of muscle stiffness. A diagnosis of DM1 was made based on the presence of classical clinical features (grip myotonia, ptosis, distal hand, leg, face, and neck weakness, hypotonia and hypersomnolence). Genetic testing revealed 750–850 CTG repeats in *DMPK*, consistent with DM1. There was no known family history of DM1 at the time of diagnosis, however, her father and sister were subsequently determined to be affected clinically and confirmed with molecular diagnosis. There was no history of cardiomyopathy, coronary artery disease, valvular heart disease or arrhythmia. Initial cardiac screening with transthoracic echocardiogram and electrocardiogram were normal. She did not present with obesity, did not smoke, and had no history of diabetes, or dyslipidemia. There is no disease-modifying treatment available for DM1. A timeline is shown in Figure 1.

The proband subsequently became pregnant at age 23. She developed mild dyspnea in the 7th month of pregnancy. Repeat transthoracic echocardiogram at 38 weeks gestation demonstrated an ejection fraction of 35–40% [normal > 55% (15)]. She was admitted to the high-risk obstetrical unit at 39 weeks gestation due a non-reassuring fetal heart rate and worsening dyspnea. She had a spontaneous vaginal delivery at 39 + 3 weeks, complicated by postpartum hemorrhage requiring transfusion. Her child was healthy. She was diagnosed with peripartum cardiomyopathy but was otherwise feeling well and discharged home with metoprolol to be followed by cardiology as an outpatient.

**FIGURE 2:**
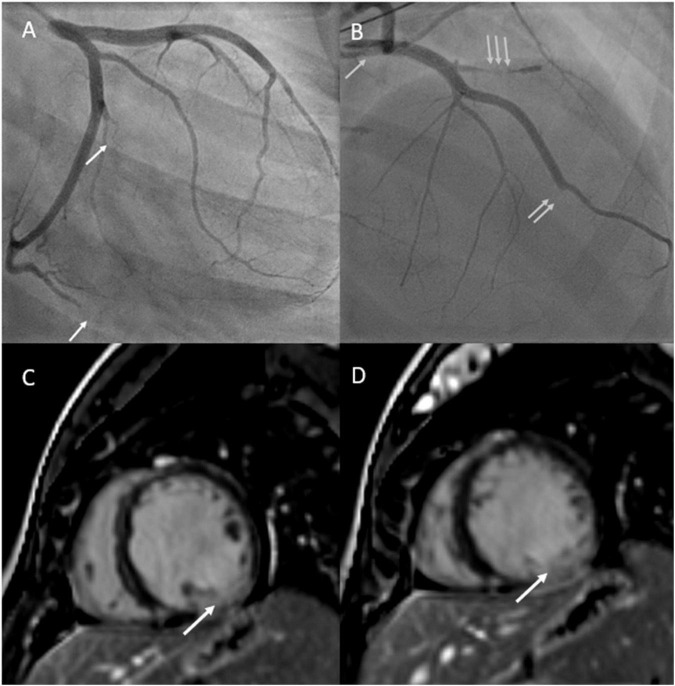
**(A)** Depicts cardiac angiogram demonstrating occluded cardiac marginal arteries (arrows). **(B)** Depicts cardiac angiogram demonstrating dissected left main coronary artery (single arrow), occluded left anterior descending artery (double arrow), and occluded diagonal artery from the left anterior descending artery (triple arrow). **(C,D)** Reveal cardiac MRI with gadolinium enhancement with phase sensitive inversion recovery showing basal to mid transmural late gadolinium enhancement in the inferior and inferolateral walls (left circumflex artery territory) secondary to spontaneous coronary artery dissection.

Five days postpartum, she developed acute retrosternal chest pain radiating to the jaw and left arm. In the emergency room, electrocardiogram demonstrated inferior lead ST elevation and she was immediately taken to the cardiac catheterization lab. Cardiac catheterization demonstrated multivessel spontaneous coronary artery dissection (SCAD) involving the ostial left main artery, first diagonal artery, obtuse marginal branches (M2 and M3 branches) and distal left anterior descending coronary artery (Figure 2). The patient had a cardiac arrest during the procedure, requiring cardiac resuscitation, intubation, and vasopressors for hemodynamic support. She stabilized rapidly and was able to be extubated within 24 h. She was started on guideline directed medical therapy for heart failure including angiotensin converting enzyme inhibitor, beta-blocker, and mineralocorticoid receptor antagonist and aspirin for SCAD. Repeat echocardiogram demonstrated a left ventricular ejection fraction of 20% with left ventricular dilatation, and mild mitral and tricuspid regurgitation. Cardiac magnetic resonance imaging confirmed left ventricular dilatation with severe left ventricular dysfunction, thin linear mid-wall delayed gadolinium enhancement in the septum, and focal transmural late gadolinium enhancement at the mid to base left ventricular inferior and inferolateral walls with hypokinesia secondary to SCAD (Figure 2). Ultrasound demonstrated left internal jugular vascular line associated thrombus and she was treated with apixaban for 3 months. She improved rapidly, was ambulant and discharged home 12 days later and remained clinically stable for 5 months.

**FIGURE 3:**
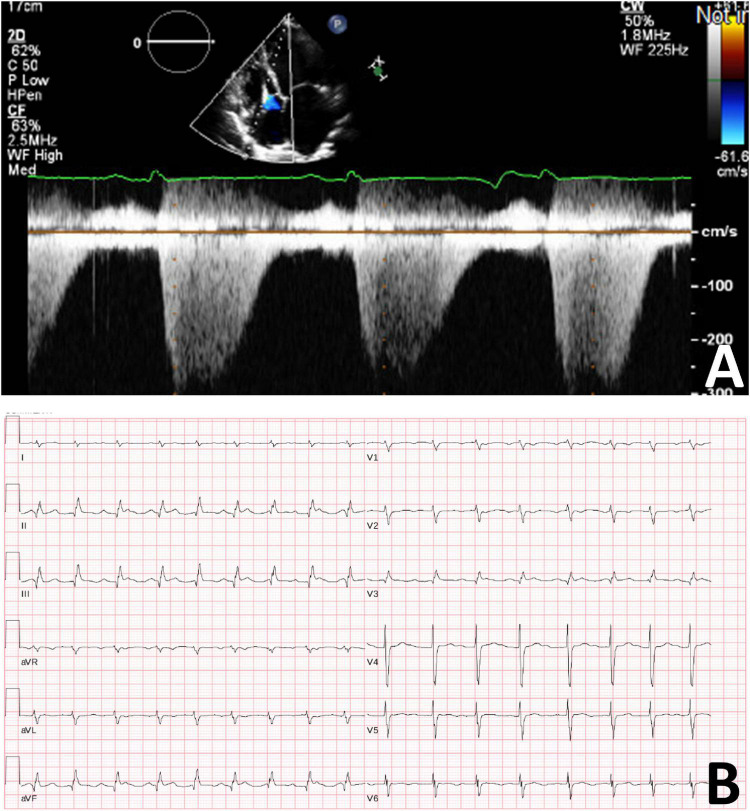
**(A)** Transthoracic cardiogram demonstrating severely reduced LV function with severe biatrial enlargement. **(B)** Electrocardiogram demonstrating sinus tachycardia with second degree A-V block (Mobitz 1).

After several months, the patient discontinued her medications (aspirin, spironolactone, and perindopril), as she was concerned that these medications were causing abdominal pain. She acutely declined and she was readmitted to hospital with nausea, abdominal pain, orthopnea, and paroxysmal nocturnal dyspnea with a diagnosis of heart failure. Transthoracic echocardiogram demonstrated worsening cardiac function with severe global hypokinesis of the left ventricle, mild to moderately reduced right ventricular systolic function, severe functional mitral regurgitation, moderate tricuspid regurgitation and pericardial effusion (Figure 3). Given persistent severe low left ventricular ejection fraction, a dual chamber implantable cardioverter defibrillator was implanted for primary prevention. However, she deteriorated rapidly, developed further cardiogenic shock, and had multiple cardiac arrests with pulseless electrical activity. She required extracorporeal membrane oxygenation, cardiac support with a miniaturized ventricular assist pump/left ventricular assistive Impella device with anticoagulation, and renal replacement therapy. She had several complications including renal failure and septicemia and developed pneumonia with computerized tomography chest demonstrating confluent consolidation in the upper and lower lung lobes. Computerized tomography abdomen showed severe congestive hepatopathy and bowel ischemia. Unfortunately, she also developed multiple large epidural and subdural hematomas and cerebral edema with worsening neurological function. Care was withdrawn due to poor neurological and cardiac function with multiorgan failure. No autopsy was performed.

## Discussion

Cardiac involvement is prevalent in DM1, occurring in approximately 80% of patients (16). Although arrhythmias and conduction defects are more common in DM1 patients, dilated cardiomyopathy has been reported (16). Larger CTG repeat expansions have been associated with a greater risk of left ventricular dysfunction, conduction defects, supraventricular arrhythmias, and sudden death (17). Women with DM1 are at an increased risk of pregnancy complications, including death (11).

Peripartum cardiomyopathy is defined as heart failure secondary to left ventricular systolic dysfunction (left ventricular ejection fraction < 45%, 45–50% on occasion (18)) without an identifiable etiology that occurs in the last month of pregnancy or within 5 months post-delivery (19). The incidence of peripartum cardiomyopathy widely varies geographically and is estimated to be between 1/900 and 1/4,000 live births in the United States (20). Complications of peripartum cardiomyopathy include thromboembolism, cardiogenic shock, arrhythmias, cardiac arrest, and sudden death (20). If treatment for peripartum cardiomyopathy is started rapidly, patients may have a partial or full recovery of cardiac function but remain at increased risk of relapse, particular with subsequent pregnancies (21). Peripartum cardiomyopathy management includes standard heart failure treatment and bromocriptine (a prolactin inhibitor) with thrombosis prophylaxis or anticoagulation (21). Bromocriptine was not given for this patient due to internal jugular thrombosis. Standard heart failure treatment, including angiotensin-converting enzyme inhibitors and beta-blockers, is strongly recommended for the treatment of dilated cardiomyopathy in neuromuscular diseases (4). A pacemaker is indicated in case of bradycardia or atrioventricular blocks, whereas symptomatic ventricular arrhythmias may require an implantable cardioverter defibrillator (4). Cardiac transplantation may be considered in motivated, ambulant patients with advanced heart failure and relatively good neuromuscular prognosis (4). Unfortunately, the patient presented in this report had developed severe multiorgan dysfunction and was not a candidate for transplant.

SCAD is a non-iatrogenic, non-traumatic and non-atherosclerotic intramural hemorrhage, causing separation of the coronary arterial wall (22). This intimal tear or spontaneous hemorrhage results in a false lumen with intramural hematoma that can compress the true lumen, causing myocardial ischemia or infarction (23). SCAD accounts for up to 35% of myocardial infarctions in women under 50 years of age and can cause cardiac arrest, myocardial infarction, or death (23).

SCAD has been associated with postpartum status, multiparity, arteriopathies, connective tissue disorders, systemic inflammatory conditions, emotional distress, and up to 86% of patients have fibromuscular dysplasia (24, 25). Pregnancy-associated SCAD can occur in the antepartum or postpartum period and is believed to be caused by the hormonal and hemodynamic changes of pregnancy (22). While early research suggested that SCAD is commonly associated with pregnancy, more recent studies have shown that pregnancy-associated SCAD represents < 5% of SCAD cases (18). Apart from being postpartum, our patient was not found to have any underlying conditions known to be associated with SCAD. Vascular imaging did not demonstrate fibromuscular dysplasia and she did not have clinical evidence of a connective tissue disorder or systemic inflammatory condition. Her underlying diagnosis of DM1 raises the possibility of an association with SCAD. While there have been no previous reports of SCAD in peripartum DM1 patients, DM1 is associated with vascular dysfunction, including systemic reductions in blood pressure (26, 27), increased susceptibility to orthostatic hypotension (28), lower coronary reserve (29), and thinner capillary basement membranes (30). It is conceivable that DM1-related vascular dysfunction, pregnancy-induced changes in cardiovascular function, and the exertional stresses of labor and delivery could place DM1 patients at elevated risk of SCAD in the peripartum period.

This is the first reported case of SCAD, and the second reported case of fatal peripartum cardiomyopathy in a DM1 patient. As the etiology of SCAD is thought to be multifactorial, there may have been several precipitating factors in this case, including postpartum hormonal status and cardiomyopathy. In most cases, conservative management of SCAD is preferred as the coronary artery intimal tear has been shown to heal spontaneously (23). Medical management of SCAD includes antiplatelets and beta-blockers. Revascularization is usually reserved for patients with ongoing ischemia or hemodynamic instability (23).

The previous report by Fall et al. (14) described one patient with DM1 who developed severe diuretic-resistant cardiomyopathy (ejection fraction 20%) and underwent dialysis for treatment of pulmonary edema prior to cesarian delivery. The patient clinically improved over 2 months with resolution of dyspnea and peripheral edema. However, she died suddenly 8 weeks postpartum from cardiac arrest (14). The combined rarity of DM1, SCAD, and severe peripartum cardiomyopathy coupled with the absence of DM1-specific treatments for heart failure were diagnostic and therapeutic challenges in the present case. This report serves to alert clinicians to the potential risk of severe cardiac disease and SCAD in DM1 patients. It also offers a strategy to increase our understanding of, and potentially mitigate, risks to DM1 patients in the future.

Physicians caring for women with suspected or proven DM1 should offer counseling and be alerted to the risk of cardiac complications with pregnancy. In addition to routine baseline cardiac function studies, any symptomatology suggestive of heart failure should prompt further dedicated cardiologic assessment. At present, there are no DM1-specific treatments. Patients who develop a cardiomyopathy are likely to benefit from early institution of standard heart failure management to prevent deterioration in cardiac function and reduce the risk of heart failure. Further research and ongoing enrollment in DM1 patient registries are required to better define the incidence of peripartum cardiomyopathy and SCAD, as well as the best therapeutic strategy for this unique clinical challenge to the growing field of cardio-obstetrics.

## Data Availability Statement

The original contributions presented in the study are included in the article. Further inquiries can be directed to the corresponding author.

## Ethics Statement

Ethical review and approval was not required for the study on human participants in accordance with the local legislation and institutional requirements. The legal representatives of the patient have provided written informed consent in support of this publication and for any potentially identifiable images or data included in this article.

## Author Contributions

GB and JW-C curated the patient data and drafted the initial manuscript. All authors reviewed the literature and edited the manuscript for scientific content.

## Conflict of Interest

The authors declare that the research was conducted in the absence of any commercial or financial relationships that could be construed as a potential conflict of interest.

## Publisher’s Note

All claims expressed in this article are solely those of the authors and do not necessarily represent those of their affiliated organizations, or those of the publisher, the editors and the reviewers. Any product that may be evaluated in this article, or claim that may be made by its manufacturer, is not guaranteed or endorsed by the publisher.
